# Lidocaine’s Ineffectiveness in Mitigating Lipopolysaccharide-Induced Pain and Peristaltic Effects in Horses

**DOI:** 10.3390/ani14213147

**Published:** 2024-11-02

**Authors:** Lara Nunes Sousa, Isabella Caixeta Winter, Diego Duarte Varela, Eduarda Zancanaro Luvison, Juan Felipe Colmenares Guzmán, Ana Moutinho Vilella Machado, Renata Diniz Vilela Figueiredo, Gabriel Tavares Pena, Ana Clara Silva dos Santos, Rafael Resende Faleiros, Armando de Mattos Carvalho

**Affiliations:** 1 EQUINOVA Research Group, Department of Veterinary Medicine and Surgery, Veterinary School, Federal University of Minas Gerais (UFMG), Belo Horizonte 31270-901, Brazil; bella_winter@hotmail.com (I.C.W.); faleirosufmg@gmail.com (R.R.F.); armandodvm@gmail.com (A.d.M.C.); 2 Veterinary School, Federal University of Minas Gerais (UFMG), Belo Horizonte 31270-901, Brazilana.ufmg.vet@gmail.com (A.M.V.M.); anaclarasantos.vet@gmail.com (A.C.S.d.S.); 3 Veterinary Medicine, Pontifical Catholic University of Minas Gerais, Belo Horizonte 30140-002, Brazil; renatadinizv@gmail.com; 4 CNPq Research Fellow, National Council for Scientific and Technological Development (CNPq), Brasília 71605-001, Brazil

**Keywords:** prokinetic, abdominal pain, intestinal paresis

## Abstract

The present study involved seven horses in a randomized crossover clinical trial to evaluate the effect of lidocaine on horses with induced endotoxemia. Horses received intravenous lidocaine (1.5 mg/kg bolus, followed by 0.05 mg/kg bwt/min) or placebo (0.9% sodium chloride at the same manner) one hour before LPS administration (0.03 μg/kg, IV infusion over 30 min). We monitored clinic and hematologic parameters, abdominal auscultation, ultrasound, and pain over time. No relevant clinical differences existed between treatments regarding peristalsis, abdominal pain, or any other parameters before and after endotoxemia induction. These findings do not support the clinical use of lidocaine to mitigate abdominal pain and intestinal hypomotility promoted by endotoxemia in horses.

## Simple Summary

Lidocaine hydrochloride is frequently used to relieve pain and stimulate peristalsis in horses with acute abdomen undergoing exploratory laparotomy. However, there is no agreement regarding its efficacy as a prokinetic and analgesic drug in international literature. Until now, there was no controlled study evaluating how lidocaine infusion influences analgesia and intestinal kinetics in horses subjected to endotoxemia. In this study, no differences had appeared between lidocaine and saline treatments in horses subjected to the lipopolysaccharide (LPS) model regarding pain and peristalsis. The results do not support the use of lidocaine for relieving abdominal pain and paralytic ileus in horses undergoing endotoxemia.

## 1. Introduction

Intestinal hypomotility and atony are common in horses with colic syndrome or during the postoperative period, leading to complications such as gastric dilatation, gastrointestinal reflux, and endotoxin absorption [1,2,3]. These events compromise vital functions and negatively affect the prognosis of the colic patient. In this context, prokinetic medications play a crucial role in restoring intestinal motility minimizing function and reducing complications [3,4,5].

Lidocaine is established for its antinociceptive, pain-relieving, anti-inflammatory, and prokinetic properties, and is frequently administered to horses in the postoperative period of colic, especially in cases involving the small intestine [6,7]. Although considered safe, there are inconsistencies and doubts about the analgesic and prokinetic efficacy of lidocaine. The variability and low repeatability of its effects have raised concerns about the cost–benefit in prolonged treatments [8,9].

These uncertainties are supported by studies that found no statistically significant improvements in intestinal contractions or only minimal effects on specific segments [10,11,12]. Nevertheless, lidocaine remains the most used prokinetic drug in the postoperative management of colic, especially in cases involving small intestine dysfunction [6,7]. The limited number of studies examining the use of lidocaine in horses during episodes of endotoxemia highlights the complexity of physiological interactions and reinforces the need for controlled and standardized research. Thus, the objective of this study was to evaluate the effects of intravenous lidocaine infusion in horses with endotoxemia, specifically regarding the maintenance of intestinal peristalsis and abdominal analgesia.

## 2. Materials and Methods

### 2.1. Experimental Design

The experiment was conducted at the EQUINOVA Teaching and Development Center, located at Fazenda Modelo of Federal University of Minas Gerais, Pedro Leopoldo, Minas Gerais. The Ethics Committee on the Use of Animals (CEUA Protocol 146/2023) approved the study. The study involved a randomized controlled clinical trial to evaluate lidocaine and placebo treatments using an endotoxemia model. A crossover design was implemented, with the treatment order being decided by drawing lots. There was a seven-day pharmacological washout period between treatments, without identification of the treatment received, and the evaluators performed the clinical evaluations blindly.

Seven adult crossbred horses (two castrated males and five non-pregnant females), with body condition scores ranging from 5 to 6/9, a mean (±SD) age of 5.1 ± 3.4 years, and a mean body weight of 307.6 ± 51.2 kg, were used. Study horses were healthy based on clinical and hematological examinations and had no history of abdominal discomfort in the six months prior to the study. For one week before each trial, the horses remained in a collective paddock with free access to green pasture and water.

On the procedure day, each horse was transferred to an individual stall with *ad libitum* water, and a peripheral venous catheter (gauge 14) was aseptically placed in each jugular vein. At that moment, horses assigned to the lidocaine group received an intravenous bolus of a 1.5% solution (LIDOVET, Bravet Laboratory, Rio de Janeiro, Brazil) at a dose of 1.5 mg/kg given in 15 min, followed by a continuous infusion (0.05 mg/kg/min) for eight hours. The placebo group received a 0.9% sodium chloride solution at the same bolus volume and infusion rates.

One hour after treatment, horses from both groups were induced to reversible endotoxemia through the intravenous infusion of 0.03 μg/kg of lipopolysaccharide (LPS) of Escherichia coli O55:B5 (L2880, Sigma-Aldrich, San Luis, Missouri, EUA), diluted in 500 mL of 0.9% sodium chloride, which was infused over 30 min using a universal infusion pump (model IPA 112, Medevo Medical Co., Beijing, China).

**Figure 1:**
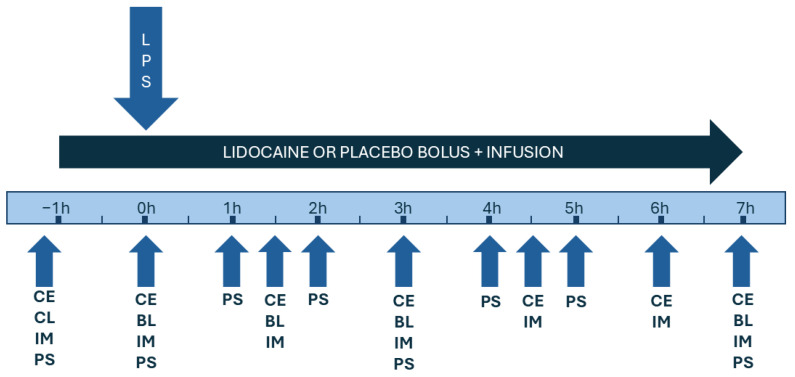
Timeline illustration indicating the treatment periods, the LPS administration time, and the timepoints for clinical examinations (CE), hematological tests (BL), intestinal motility exams (IM), and pain score assessments (PS) in horses subjected to endotoxemia and treated with lidocaine or saline solution infusions.

Horses were assessed through clinical examination (CE), hematological tests (BL), intestinal motility exams (IM), and pain scores systems (PS), immediately before treatment administration (T–1), immediately before (T0) LPS infusion, and over time as shown in Figure 1. For clinical examination, heart (HR) and respiratory (RR) rates, rectal temperature (RT), capillary refill time (CRT), and mucous membrane congestion grades (1—pale/white; 2—pink color; 3—red; 4—purple/blue; and 5—yellow) were recorded. Blood samples were taken from the jugular vein with a vacuum collection system and stored in EDTA K3 tubes. The samples were immediately refrigerated and later sent to the laboratory for blood count analysis.

Two methods assessed intestinal motility: auscultation of borborygmus and transabdominal ultrasound examination. A stethoscope (Classic III model, Littmann, Canada) facilitated the auscultation of the borborygmus intensity [13]. The score borborygmi simultaneously by two evaluations as previously described. An ultrasound machine (A6V, Sonoscape, Guangdong, China) assessed the intercostal position of the gastric caudal border, and to count intestinal contractions per minute in small and large intestinal segments.

For the ultrasonographic examination (US), trichotomy and the direct application of an alcohol solution ensured contact between the probe and the skin. Two independent researchers counted the intestinal contractions, and the result was obtained by calculating the arithmetic mean.

Pain was assessed using two score systems, the modified EQUUS-COMPASS and the EQUUS-FAP [14], as described in Appendix A. To minimize bias during pain assessment, horses remained in quiet individual stalls while DVR digital video (MHDX 1008, In-telbras, Santa Catarina, Brazil) recording system recorded their behavior, connected to a bullet camera.

### 2.2. Statistical Analysis

Data analysis utilized GraphPad Prism 10.1.2. Parametric data underwent normality testing with the Shapiro–Wilk and the Kolmogorov–Smirnov tests before two-way ANOVA assessed the effect of time, treatment, and their interaction. For non-parametric data, the Friedman test evaluated the effect of time over each treatment group, while the Wilcoxon test compared treatments in each timepoint. A significance level of *p* < 0.05 applied to all tests.

## 3. Results

**Figure 2:**
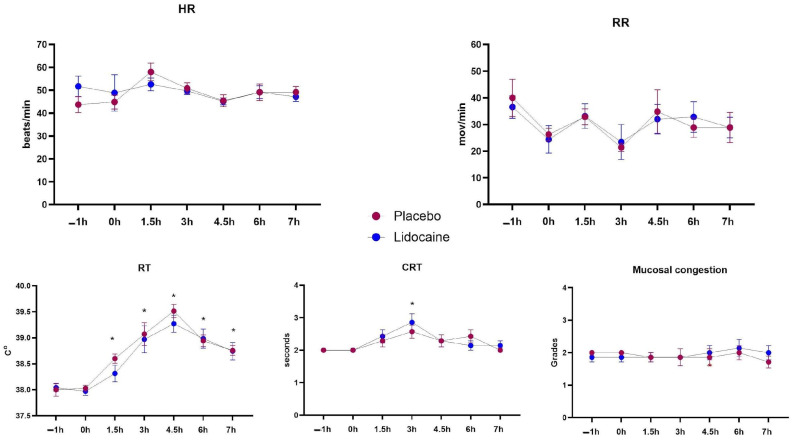
Means and standard errors of heart (HR) and respiratory (RR) rates, rectal temperature (RT), capillary refill time (CRT), and degrees of mucosal congestion in horses subjected to endotoxemia, treated by infusion of lidocaine (blue) or saline solution (red). * Differs from basal values considering both treatments (*p* < 0.05).

Data from the physical exams appear in Figure 2. A significant time effect was noted for two variables, but no treatment or interaction effects were observed in any of them. A consistent increase in RT occurred at T1.5 and continued until the end of the experimental period, when compared to baseline (T–1 and T0). Additionally, there was an increase in CRT values 3 h after T0.

**Figure 3:**
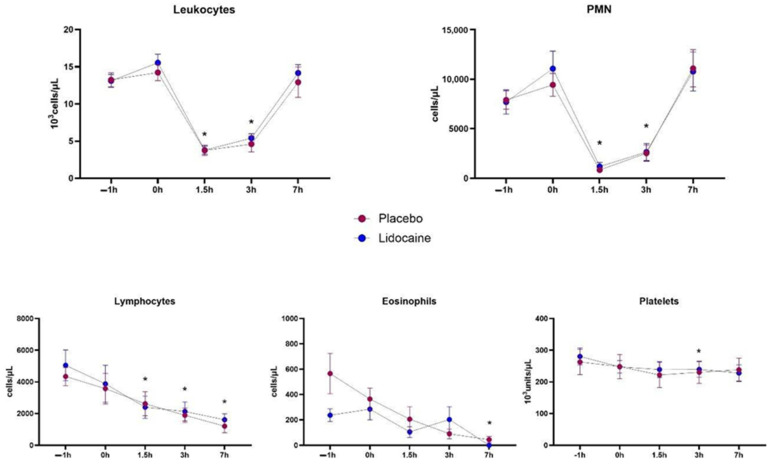
Mean and standard errors of total leukocyte, polymorphonuclear (PMN), lymphocytes, monocytes, eosinophils, and platelet counts in horses subjected to endotoxemia, treated by infusion of lidocaine (blue) or saline solution (red). * Differs from basal values considering both treatments (*p* < 0.05).

In the white blood cell counts (Figure 3), total leukocytes, polymorphonuclear cells (PMN), lymphocytes, and eosinophils showed a time effect, but there were no significant treatment or interaction effects. A decrease in total leukocytes and PMN counts occurred between T1.5 and T3. In an analogous way, there was a reduction in lymphocyte counts starting at T1.5, and eosinophils counts at T7. Platelet counts showed statistical change only at T5 in relation to T0, with no difference between groups (Figure 3).

### 3.1. Evaluation of Effects on Intestinal Motility

**Figure 4:**
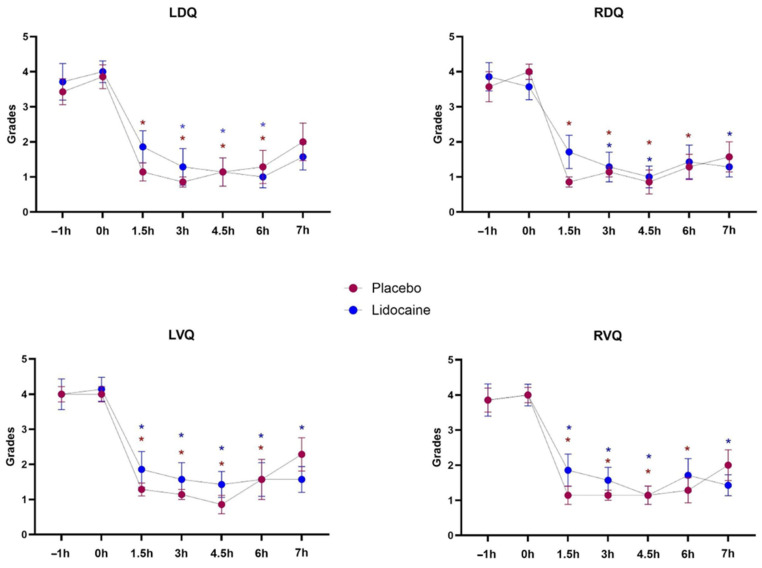
Mean and standard errors of the left dorsal (LDQ), right dorsal (RDQ), left ventral (LVQ), and right ventral (RVQ) abdominal quadrants borborygmus intensity grades in horses subjected to endotoxemia, treated by infusion of lidocaine (blue) or saline solution (red). * Differs from basal values considering each treatment (*p* < 0.05).

**Figure 5:**
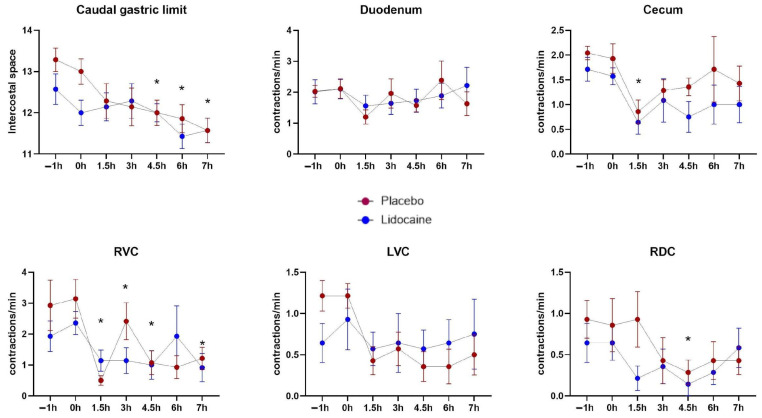
Mean and standard errors of the intercostal space of the caudal gastric edge, and the counts of contractions of the duodenum, cecum, the left ventral (LVC), right ventral (RVC), and right dorsal (RDC) colons of horses subjected to endotoxemia, treated by infusion of lidocaine (blue) or saline solution (red). * Differs from basal values considering both treatments (*p* < 0.05).

There was a reduction in the intensity of borborygmus auscultation in all abdominal quadrants of both groups after endotoxemia induction, but no statistical differences in the comparison between treatments (Figure 4). Ultrasonography detected a significant effect of time on intestinal contractions, but no relation to treatments or interaction. Decreases compared with T0h occurred in the gastric dimension starting at T4, and in the contraction frequency of the right ventral colon at T1 (Figure 5).

### 3.2. Evaluation of Effects on Abdominal Discomfort

**Figure 6:**
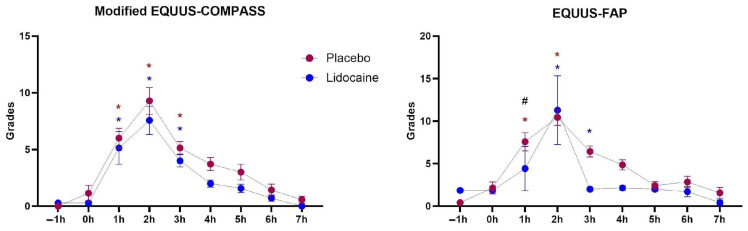
Means and standard errors of the degrees of pain intensity obtained by the EQUUS-FAP or modified EQUUS-COMPASS scores systems in horses subjected to endotoxemia, treated with lidocaine infusion (blue) or saline solution (red). * Differs from baseline in the same group (*p* < 0.05). # Differs from the other treatment at the same timepoint.

Regardless of the treatment or the employed scoring system, pain intensity increased after LPS administration from T1 to T3. Only at one timepoint (T1), a slight difference between treatments (*p* < 0.05) was observed within the EQUUS-FAP scoring system (Figure 6).

## 4. Discussion

This is the first controlled study to evaluate the analgesic and prokinetic effects of lidocaine infusion in horses subjected to endotoxemia. The crossover and blind design helped reduce variability, as horses served as their own controls, thereby enhancing the validity and precision of the results.

The infusions of lidocaine and placebo were initiated prior to the administration of LPS, adopting a therapeutic approach previously proposed [9]. This choice was based on the idea that early treatment is considered the most effective method to evaluate the efficacy of a drug in an experimental model, and to ensure that adequate plasma concentrations would be available to possibly mitigate the earliest effects of LPS administration. The action and metabolism of lidocaine are rapid. With a bolus infusion rate of 1.3 mg/kg administered over 15 min, followed by a continuous infusion rate of 0.05 mg/kg/min, plasma concentration stabilizes in approximately one hour. The metabolites produced are detectable within minutes after the infusion begins, contributing to lidocaine’s pharmacological effects [15].

The efficacy of the endotoxemia model confirms in this study based on the occurrence of classic effects over the clinical, hematological and behavioral parameters in placebo-treated horses, like hyperthermia, leukopenia and abdominal pain. These indicators receive extensive documentation in the literature for the equine species [16,17,18,19,20], and previous reports indicate that the administration of 0.03 μg/kg (IV) of LPS was sufficient to trigger a systemic inflammatory response in horses [21].

Hyperthermia consistently occurred in both groups. It started 1.5 h after LPS began administration (T0), peaked at 4.5 h and persisted until the end of the experimental period (7 h). Transient changes in CRT also appeared at 3 h after T0, attributed to hypovolemia caused by fluid loss due to diarrhea, intestinal edema, or sequestration of fluid into the lumen [22]. Unlike the studies that reported normal feces and no difference in humidity during equine experimental endotoxemia [14,19], this did not occur in this study. A small number of animals used in the study manifested pasty and diarrheal feces after endotoxemia induction, suggesting water sequestration in intestinal loops. Four placebo-treated horses showed changes in fecal consistency 1.5 h after T0, ranging from slightly pasty (*n* = 3) to diarrheal (*n* = 1) feces. In contrast with lidocaine-treated horses, only one exhibited pasty feces.

The hematological findings of this study corroborate previous observations that reported a significant reduction in the total number of circulating leukocytes associated with a decrease in segmented neutrophils and lymphocytes after LPS administration [16,18,23]. Other studies also reported leukocytosis in later stages [24], which might be detected in the present study with a longer experimental period. In a similar equine model when endotoxemia was induced by LPS intraperitoneal administration, no significant differences were observed in the levels of red blood cells, hemoglobin, hematocrit, and basophils [25].

A potential limitation of this study includes the relatively short washout period of 7 days. Although others have adopted the same administration interval [18], there is scientific evidence that pain response to LPS may decrease seven days later [17]. No information about the influence of the washout period over the peristaltic response to LPS exists, and we did not observe any significant effect in our pilot study. To minimize the possible influence of LPS tolerance over the treatments (placebo or lidocaine), we altered their sequences among individuals during the trial. Therefore, this potential bias does not affect our results.

No significant differences in peristalsis were observed between treatments by the auscultation of the abdominal quadrants. However, there was a decrease in the borborygmus intensity in all quadrants after endotoxemia induction in both groups. Our findings support those described by other authors, who also observed intestinal hypomotility as a clinical effect of endotoxemia induction [18,20] and reported a marked decrease in the frequency of bowel sounds in the upper and lower right quadrants after 75 min of endotoxemia [16].

Abdominal auscultation is considered an indirect method of motility assessment, differing from percutaneous/transabdominal ultrasonography, which allows the direct visualization of structures to assess the range and frequency of motion [26], making it a more reliable approach. In the present study, the mean frequency of contractions detected by the US after T0 were numerically inferior compared to basal values. However, the variability among individuals at each timepoint reduced the statistical power to determine whether these changes were due to the LPS effect or whether they occurred by chance.

On the other hand, a statistical decrease in gastric dimensions following endotoxemia was detected by the US. Nevertheless, this can be considered physiological, as horses did not have access to food during the observational period and there was no difference between groups. The retention of hay or green grass in the stomach can range from two to six hours [27], a period that coincides with the duration of the experiment. One study reports minimal effects of administering a bolus of lidocaine (1.3 mg/kg per 30 min) in gastric emptying [5], which could also be observed in the present study.

It has been consistently reported that systemic lidocaine administration did not affect the frequency of borborygmus between the quadrants or segments evaluated in healthy horses [10,11,12]. This is supported by both jejunal myoelectric stimulation [11] and the borborygmus score [10,12] in horses under continuous lidocaine infusion. This study endorsed these findings by demonstrating that bowel sounds did not show any significant differences with lidocaine treatment.

Other researchers have reported that lidocaine administration did not affect gastrointestinal auscultation, motility, and thickness of either the small or large intestines [26], nor did it affect the peristalsis of the small intestine and cecum, as assessed by percutaneous electrointestinography [5] in horses undergoing laparotomy. However, the frequency of duodenal contractions was reported to be increased in the lidocaine treatment group when compared to the placebo in healthy horses subjected to a 12 h fasting period [12].

Our findings did not demonstrate significant differences in the motility of any intestinal segment in horses treated with lidocaine compared to placebo. It is believed that the positive results of this therapy occur only in specific cases, in which lidocaine would be able to reduce the adrenergic sympathetic transmission of intestinal nerve impulses. Thus, in the absence of positive effects, it is necessary to consider other medications proven to be prokinetic for this purpose, such as mosapride, metoclopramide, or cisapride [5].

Significant analgesic effects of lidocaine administration were also not observed in this study. Although a lower pain score (P0.03) for lidocaine treatment was detected 1 h after LPS administration with the EQUUS-FAP scoring system, no other differences between pain intensity were detected between treatments. Also, the peak of the facial pain score occurred 2 h after T0 in both groups. In another study, the peak of pain occurred approximately 60 min after endotoxin infusion, with higher scores in this period when compared to baseline [18]. In this study, signs of abdominal pain began 60 min after T0 (endotoxin infusion) in both groups.

Both scales were validated for the assessment of acute pain caused by colic syndrome. The exclusion of physiological parameters leads to comparable results and makes it possible for owners and caregivers to use them reliably. Facial pain scores below four are considered indicative of no pain, while scores above six are associated with patients that may require surgery [4]. Placebo-treated horses exceeded this threshold from T1 to T5, and lidocaine-treated ones from T2 to T4. However, in contrast with the irrelevant analgesic effects of lidocaine infusion in this study, the use anti-inflammatory and analgesic drugs prior to LPS infusion promoted a notable reduction in the levels of pain and other endotoxemic effects compared to the placebo group [19,28].

Also supporting our findings, previous studies using other models of visceral pain did not report any relevant analgesic effects of abdominal pain promoted by the intravenous infusion with lidocaine [8,29]. There are reports that the clinical and analgesic effects of lidocaine are more evident in cases of intestinal inflammation [11]. Therefore, lidocaine could relieve peristaltic impairment specifically produced by intestinal pain and inflammation. Paralytic ileus is commonly reported in horses undergoing intestinal surgery with exacerbated bowel inflammation or ischemia, severe intestinal distension, or when intense mechanical manipulation is required. However, no improvement in pain scores was reported with lidocaine infusion in horses with induced jejunal ischemia [9].

It is believed that pain associated with endotoxemia can produce intense sympathetic (adrenergic) nerve activity and increases in circulating catecholamines, which result in reduced propulsive intestinal motility [30]. However, in this study, lidocaine treatment failed to produce any measurable antinociceptive or anti-inflammatory effect capable of ameliorating the LPS-induced intestinal paresis. Corroborating our findings, a similar lidocaine therapy was not effective in treating inflammation in horses with induced laminitis [31].

Based on our findings, the hypothesis of this study is null, which sought to evaluate the beneficial effects of lidocaine administration in LPS-induced hypomotility and pain in horses. However, it is worth considering that the experimental endotoxemia created by LPS infusion is not the same as an acute abdomen endotoxemia, when many other pathogen-associated molecule patterns (PAMPs) and other associated factors, like damage-associated molecular patterns (DAMPs), hypovolemia, and metabolic and electrolyte imbalances may interfere with visceral pain and peristalsis dysfunctions. Considering these limitations, it is premature to completely discard the use of lidocaine infusion in the therapy of pain and paralytic ileus in equine clinical cases.

## 5. Conclusions

Lidocaine infusion fails to mitigate the abdominal pain and hypomotility induced by the LPS endotoxemia model in horses. These findings do not support the use of lidocaine to mitigate abdominal pain and intestinal hypomotility promoted by LPS in horses. However, it is premature to completely discard its therapeutic use due to other factors that may be involved in a clinical scenario.

## Data Availability

The data that support the findings of this study are not publicly available as it contains information that could compromise the privacy of research participants. The article is an expanded and revised version of a poster with preliminary data titled “Ação da lidocaína intravenosa na motilidade intestinal e na analgesia de equinos com endotoxemia induzida” which was presented at the Annual ABRAVEQ Conference in Campinas, São Paulo, Brazil, in 2024. As of the time of submission, the conference proceedings have not yet been made available, which justifies the absence of this reference in the article. This is the first full submission of the work.

## Appendix A

Researchers monitored and assessed abdominal discomfort in horses using two scales developed by Van Loon and Van Dierendonck [14]: the Equine Utrecht University Scale for Composite Pain Assessment (EQUUS-COMPASS) and the Equine Utrecht University Scale for Facial Assessment of Pain (EQUUS-FAP). The EQUUS-FAP assesses pain based on nine facial parameters, while the EQUUS-COMPASS uses fourteen parameters, covering physical and well-being aspects, with a special focus on visceral pain. In this study, it was decided to exclude physical parameters from the EQUUS-COMPASS for methodological adjustments.

Digestive abdominal auscultation utilized the Borborygmi Score proposed by Boscan et al. [10], classifying the sounds from zero to five based on type and frequency. The evaluation considered four quadrants: right and left dorsal, and right and left ventral.
